# The Effect of Astaxanthin on Quality of the Common Cuttlefish (*Sepia officinalis*) Marinade

**DOI:** 10.1002/fsn3.71724

**Published:** 2026-04-05

**Authors:** Pınar Baldemir, Şükran Çaklı

**Affiliations:** ^1^ Department of Fish Processing Technology, Fisheries Faculty University of Ege Izmir Turkey

**Keywords:** antioxidant capacity, astaxanthin, bioactive compounds, food quality, functional foods, microalgae, sustainable food

## Abstract

In this study, cuttlefish ( *Sepia officinalis* ), a member of the *Cephalopoda* class, was used as the raw material, since its mantle is a species rich in total lipids in fatty acids and polyunsaturated fatty acids. After boiling the thawed fish, marinades were prepared according to the groups in the method section. Astaxanthin pigment, extracted from *Haematococcus pluvialis* microalgae, was added to the marinade alongside garlic powder and parsley for their antioxidant properties, and changes in quality parameters were observed in the groups produced with different formulations. Sensory (appearance, smell, taste, texture), microbiological (mold‐yeast, lactic acid bacteria, total mesophilic aerobic bacteria, total psychrotrophic bacteria, coliforms, fecal coliforms, *Staphylococcus aureus* , *Enterobacteriaceae*), and chemical analyses (pH, TVB‐N, TBARS, Crude Oil, Crude Ash, Moisture, Acid, Salt, Color) were repeated at 1‐month intervals over 4 months. Antioxidant capacity (DPPH scavenging activity) remained highest in the combined astaxanthin‐garlic powder‐parsley group (ASM) with 49.3% retention on Day 120, while lipid oxidation was lowest in the astaxanthin‐only group (TBARS: 6.88 μmol MDA/kg). Fatty acid composition and antioxidant capacity determination analyses were performed in 2 periods, at the beginning and end of the storage period. The effect of storage duration on product quality was evaluated in the experimental groups, which were stored at 4°C immediately after production, was determined. Statistically significant changes were observed in chemical, physical, microbiological and sensory values (*p* < 0.05). For all parameters, the results in all groups remained below the consumability threshold during the study period.

## Graphical abstract

The effects of astaxanthin sourced from *Haematococcus pluvialis* on quality parameters such as oxidative stability, microbiological safety, and sensory acceptability were investigated by adding it to cuttlefish ( *Sepia officinalis* ) marinade, during storage period.

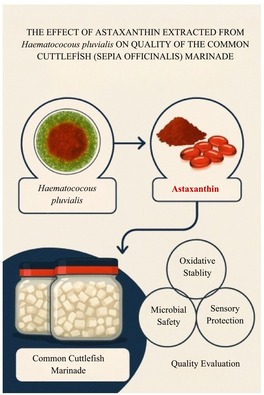

## 1 Introduction

Global per capita consumption of aquatic animal foods has steadily increased, reaching 20.7 kg in 2022 compared with 9.1 kg in 1961, more than doubling over this period (FAO 2024). Among seafood, cephalopods such as *Sepia officinalis* are valued for their nutritional profile, particularly their high lipid and polyunsaturated fatty acid (PUFA) content, which supports cardiovascular and overall health (Steur et al. 2021). However, spoilage and oxidative degradation cause substantial economic losses to the seafood industry (Hussain et al. 2021).

Microalgae have gained attention as sources of bioactive compounds—including carotenoids and PUFAs—with applications in the food, cosmetic, and pharmaceutical industries. These bioactives have demonstrated antioxidant, anti‐inflammatory, antiviral, antibacterial, antifungal, and antitumor activities (Sun et al. 2023). Among them, astaxanthin is a fat‐soluble carotenoid widely distributed in microalgae, yeasts, bacteria, plants, and crustaceans, but occurs in the highest concentrations in *Haematococcus pluvialis* (up to 40 mg/kg dry weight, six times higher than *Chlorella zofingiensis*) (Dang and Yu 2024). Its role as a natural antioxidant in improving seafood stability and shelf life has been demonstrated (Liu et al. 2022; Liu et al. 2020). A bibliometric analysis reported 1877 publications on *H. pluvialis* and 5921 on astaxanthin, underscoring growing global interest since 2020 (Mularczyk et al. 2020).

Unlike prior research on species such as anchovy, trout, or mackerel (Carballo et al. 2019; Shahidi and Zhong 2019), this study applies astaxanthin supplementation to cuttlefish, a PUFA‐rich cephalopod rarely examined in functional marinades. The antioxidant was not encapsulated but directly extracted from GMP‐standard softgel capsules containing *H. pluvialis* ‐derived astaxanthin and combined with parsley and garlic powder—ingredients known for antimicrobial and phenolic‐rich profiles. This synergistic combination has not been reported in peer‐reviewed studies on cuttlefish preservation.

Preliminary sensory trials determined optimal acid, salt, and spice levels to align the formulation with consumer preference. The study focuses on oxidative stability, microbial safety, and sensory quality—critical for cephalopods given their susceptibility to early deterioration (García‐Soto et al. 2022; Balti et al. 2017). By targeting both oxidative and microbial spoilage, astaxanthin was combined with traditional marinade constituents (Shahidi and Zhong 2019; Miller et al. 2021). Rising consumer demand for clean‐label foods and restrictions on synthetic additives further highlight the need for natural preservation strategies (Yang et al. 2021; Ribeiro et al. 2021). This research aims to enhance sustainability, reduce food waste, and improve chemical, microbiological, and sensory stability in seafood storage (Duarte et al. 2023).

A systematic literature review of high‐impact studies was performed, focusing on the antioxidant and antimicrobial activities of natural compounds, especially astaxanthin, in seafood preservation (Carballo et al. 2019; Bingol et al. 2022; Chen et al. 2023; Zhu et al. 2022; Yousef and Balasubramaniam 2020; Hossain et al. 2017; Ibrahim et al. 2022). Additional works on sensory, microbiological, and chemical characterization under storage and marination effectiveness were also considered (Chen et al. 2023; Ganesan et al. 2020). Based on this evidence, we developed a novel fish marination formulation that integrates recent preservation technologies with sustainable, health‐oriented concepts, expanding the application of natural preservation solutions in the seafood industry (Huang et al. 2020).

## 2 Materials and Methods

### 2.1 Material

During the whole study, 20 kg of cleaned cuttlefish ( *S. officinalis* Linnaeus, 1758), stored at −18°C and frozen for 1 month, was used as raw material. The astaxanthin pigment used in this study was extracted from GMP‐certified softgel capsules containing 4 mg of natural astaxanthin derived from *H. pluvialis* (AstaReal). The softgel capsules were pierced, and the lipid‐based astaxanthin solution was directly aspirated and added to the marinade mixture without further encapsulation. In preliminary sensory trials, various acid, salt, garlic powder, and parsley concentrations were tested. Based on hedonic ratings by five trained panelists, the most preferred formulation was selected for subsequent storage experiments. This formulation included 12% NaCl, 6% citric acid, garlic powder (0.5%, w/w), parsley (0.5%, w/w), and astaxanthin equivalent to 4 mg per 100 g product (Josipović et al. 2015; Bingol et al. 2022). We selected a brine formulation of 6% citric acid (w/v) and 12% NaCl, which is analogous to the traditional marinade process reported by the FAO and validated in more recent fish marination studies (FAO 1999). The final study design consisted of four groups: CONT (control), AST (with astaxanthin), SM (spices only), and ASM (combined). Each treatment group consisted of three biological replicates, with all analyses repeated in triplicate (*n* = 3).

### 2.2 Method

#### 2.2.1 Processing Method

The frozen cuttlefish was thawed 24 h in advance by placing it in a low temperature storage compartment at 0°C–4°C. After the thawed cuttlefish was washed with cold tap water at 4°C, it was boiled at 100°C for 15 min (Özoğul 2008).

Brine ratio was 6% citric acid (C_6_H_8_O_7_) (JT Baker, 002520004), 12% salt (NaCl) (Merck, K 30005704) in 1 L plastic barrels a 1.5:1 cuttlefish‐to‐brine ratio, and the material was matured in cold storage at 4°C for 24 h until the marination was completed in the flesh structure (until the cuttlefish did not show resistance to cutting with a knife).

A total of 20 kg of cleaned cuttlefish mantle (approx. 75–90 individuals) was used in this study, evenly divided across four treatment groups (approx. 5 kg each). All fish were wild‐caught in the Aegean Sea and frozen at −18°C for 1 month prior to use.

Marinade solutions were prepared fresh for each replicate, and each biological replicate comprised one 1 L sealed glass jar containing 500 g of cuttlefish and 500 mL of the respective marinade.

In this study, the formulation that was most appreciated in terms of sensory was selected and used after preliminary testing by the subjects. The marinades, which were determined to be mature by sensory observation, were taken out of the solution, filtered, and systematically prepared in the storage containers (150 mL glass jars) to be used in the main storage containers, each with a 1:1 cuttlefish‐to‐solution ratio and closed in an airtight manner. Four marinade formulations were prepared by adding garlic powder, parsley, and astaxanthin pigment, a valuable compound extracted from *H. pluvialis* microalgae.

All analyses for all prepared samples stored at 4°C were conducted in triplicate at 1‐month intervals throughout the storage period.

#### 2.2.2 Analysis Methods

##### 2.2.2.1 Product Yield Calculation

For the product yield of the obtained products, the formula given below was used after being modified (Rora et al. 1998; Cardinal et al. 2001).

$$\text{Product yield}\;\left(\%\right):\left(\frac{\text{total meat weight before processing}-\text{total meat weight after processing}}{\text{total meat weight before processing}}\right)*100$$

##### 2.2.2.2 Food Composition Analyses

###### 2.2.2.2.1 Fat Analysis

Fat analysis was performed according to Blig and Dyer (1959). A 20 g sample was mixed with 100 mL of methanol–chloroform solution (1:2, v/v). Then, washing was done with 20 mL of methanol–chloroform. The mixture was filtered through pre‐weighed filter paper into a tared flask. After the completion of the filtration process, the flasks containing the samples to which 20 mL of 0.4% CaC1_2_ was added are closed and kept in the dark for 1 day. Phase formation in the samples was observed after 24 h. The resulting balloon content was taken into the separation funnel and the process was continued. The upper phase was removed from the environment by taking the lower phase into the same balloon. The balloon containing the lower phase was taken into the rotary evaporator. After the separation of the oil in the balloon was taken into the rotary evaporator, the balloon was removed from the apparatus and kept in an oven at 105°C for 1 h. Immediately after this process, the balloon was cooled in a desiccator and the final weight was measured.

$$\mathrm{Fat}\;\text{content}\;\left(\%\right):\text{Last weight}-{\text{First weight}}^{*}100/\text{Sample weight}$$

###### 2.2.2.2.2 Moisture Analysis

Moisture analysis was performed according to Ludorf and Meyer (1973). Sterile Petri dishes were dried at 105°C for 1 h. A cooling process was applied to the desiccator for 30 min. Then, a 5 g sample was taken from the material. The empty tare of the first petri dish was taken and then the sample was added, and the weight was measured again. The result can be obtained when the material was rested in the oven for 3–4 h.

$$\text{Moisture content}\;\left(\%\right):\text{Last weight}-{\text{First weight}}^{*}100/\text{Sample weight}$$

###### 2.2.2.2.3 Ash Analysis

Ash analysis was performed according to Association of Official Analytical Chemists (1984). The crucible, which was kept in a muffle furnace at 550°C for 1 h, was subjected to a cooling process in a desiccator. Then, the tare of the crucible was taken. A sample of 2 g was weighed and kept in a muffle furnace for 3–4 h until it turns into cigarette ash. The sample to be analyzed for ash was cooled in a desiccator and the weight was measured again.

$$\mathrm{Ash}\;\text{content}\;\left(\%\right):\text{Last weight}-{\text{First weight}}^{*}\;100/\text{Sample weight}$$

##### 2.2.2.3 Fatty Acid Composition Analysis

Fatty acids present in anchovy and its oil were examined using a Perkin Elmer AutoSystem XL Gas Chromatograph equipped with an SP‐2330 capillary column and a flame ionization detector (FID). A fused silica capillary column with film thickness dimensions of 30 m × 0.25 mm × 0.20 μm enabled the separation of fatty acid methyl esters. The oven temperature was initially set at 120°C for 2 min, then increased to 220°C at a rate of 5°C per minute. The temperature was then kept constant at 5°C/min for 15 min. Injector and detector temperatures were set at 240°C and 250°C, respectively. Helium gas served as the carrier at 10 psi with a split ratio of 1/50, while air and hydrogen were maintained at flow rates of 338 and 45 mL/min, respectively. Fatty acids were determined by comparing the retention times of fatty acid methyl esters (FAME) to times in a standard 37‐component FAME mixture (Supelco‐Catalog No: 18919‐1 Amp). Results are reported as the percentage of each fatty acid relative to total fatty acids. Each analysis was performed in triplicate and results were expressed as the mean percentage of the total FAME area (IUPAC 1979).

##### 2.2.2.4 Shelf Life Analysis

###### 2.2.2.4.1 pH Analysis

pH value measurement (with HANNA model Microprocessor pH meter) was made in brine and fish meat. Measurements were made on fish meat by diluting it in a ratio of (1:1) (Ludorf and Meyer 1973).

###### 2.2.2.4.2 Total Volatile Basic Nitrogen (TVB‐N) Analysis

TVB‐N was performed according to Antonacopoulos and Vyncke (1989). A 10 g fish sample mixed with a small amount of water was thoroughly homogenized. Then, this fish meat—water homogenized mixture was transferred to the Antona steam distillation unit reaction vessel. Before the vessel was placed in the preheated steam generator, 2 g MgO and 2–3 drops of antifoam emulsion were added and distillation was started without delay. On average, 10 mL of steam was formed per minute. The distillate was kept in a vessel filled with 10 mL of 3% boric acid, 8 drops of Tachyro indicator and approximately 100 mL of distilled water. The distillation period was approximately 12 min. During 10 min of this period, the tip of the cooler was immersed in the receiver and excluded from the distillate during the remaining 2 min. The distillate was titrated with 0.1 M hydrochloric acid or 0.05 M sulfuric acid until a neutral gray point was formed.

$$\mathrm{TVB}-N\;\left(\mathrm{mg}/100\;g\right)=100-\left(\text{acid consumption}\times 14\right)$$

###### 2.2.2.4.3 Thiobarbituric Acid Reactive Substance (TBARS) Measurement

The method given by Lemon (1975) was used to determine the TBARS unit value. One gram samples were taken from the samples cut into small pieces and 6 mL of TCA solution (7.5% TCA, 0.1% EDTA, 0.1% propyl gallate) was added. After this mixture content was homogenized in the Ultra‐Turrax device for 15–30 s, filtration was provided using Whatman 1 filter paper. Three milliliter of this extract obtained as a result of the process was taken and 3 mL of TBA solution was added. Then, incubation was applied in a water bath at 95°C–100°C for 40–45 min. Cooled samples (5–10 min at room temperature) were read in a spectrophotometer at a wavelength of 532 nm. In the light of the findings obtained, the value obtained from the standard curve created was applied to the formula and the TBARS values were expressed as μmol malondialdehyde/kg of tissue was calculated in this way (Alak 2011).

###### 2.2.2.4.4 Color Analysis

Color analysis was measured using a DRLANGE Spectro‐pen brand color determination device. In color evaluation, the “L*” variable measures brightness. “a*” indicates red (+) and green (−) axes, “b*” indicates yellow (+) and blue (−) axes. Ten different readings were made in each sample (Schubring 2003).

###### 2.2.2.4.5 Acidity Analysis in Brine

Determination of acidity in brine content: 2 g of brine is placed in a 300 mL Erlenmeyer flask. This 2 g of brine in the Erlenmeyer flask is diluted with 100 mL of distilled water. The Erlenmeyer flask is covered with glass suitable for the dimensions of the flask and, if necessary, a weight is placed on it to provide additional precautions. The flask is heated to near boiling, then left to stand for 10 min. The content is left for approximately 10 min, and after waiting for it to cool, it is titrated with 0.1 N NaOH by adding a few drops of phenolphthalein (Ludorf and Meyer 1973; Schormuller 1968a).

For citric acid:

$$\text{Acid content}\;\left(\%\right):0.1\;N\;{\text{NaOH consumption}}^{*}\;0.7/2$$

###### 2.2.2.4.6 Acidity Analysis in Fish Meat

Determination of acidity in fish meat: 20 g homogenized fish meat raw material is mixed with 100 mL heated distilled water for 2–5 min. Then, the total volume was brought to 250 mL with distilled water. After these processes, the content is filtered with a Falten filter. Three drops of 1% phenolphthalein are added to the 20 mL Falten filter filtered content and titrated with 0.1 N NaOH.

For citric acid:

$$\text{Acid content}\;\left(\%\right):0.1\;N\;{\text{NaOH consumption}}^{*}\;0.7^{*}\;12.5/\text{Sample weight}$$

###### 2.2.2.4.7 Analysis of Salt Content in Brine

Determination of salt (NaCl) level in brine: 5 g of brine is transferred to a 250 mL volumetric flask. Then, distilled water is added up to the volumetric flask mark. Ten milliliter (*F*—25) of this content is taken and added by dropping onto 5 drops of phenolphthalein solution. The solution is neutralized with 0.1 N NaOH until it gives a color close to pink (which can remain for at least 30 s). Then 5 drops of K_2_CrO_4_ (5%) are added and subjected to titration with 0.1 N AgNO_3_ until a permanent red‐brown color is formed (Ludorf and Meyer 1973; Schormuller 1968a).

The amount of filtrate to be taken depends on the estimated salt amount; 50 mL (*F* = 5) up to 3% salt amount, 25 mL (*F* = 10) up to 3%–10% salt amount, 10 mL (*F* = 25) up to 10%–20% salt amount.

$$\begin{array}{c} \text{Salt content}\;\left(\%\right):\text{Used}\;0.1\;N\;{\text{AgNO}}_{3}\;{\text{consumption}}^{*}\;0.525^{*}\\ \;F/\text{Sample weight} \end{array}$$

###### 2.2.2.4.8 Analysis of Salt Amount in Fish Meat

Determination of salt amount in fish meat: After neutralization with NaOH, 3 drops of 10% K_2_CrO_4_ are added to the content and subjected to titration with 0.1 N AgNO_3_ until the color change occurs in the content.

The salt and acidity levels were measured in the brine content and fish meat content by taking samples from Karl (1994). The measurements were made on the 1st day of maturation for the brine, and regularly every month for the fish meat content after they were placed in jars.

$$\begin{array}{c} \text{Salt content}\;\left(\%\right):\text{Used}\;0.1\;N\;{\text{AgNO}}_{3}\;{\text{Consumption}}^{*}\;0.5845^{*}\\ \;12.5/\text{Sample weight} \end{array}$$

##### 2.2.2.5 Antioxidant Capacity Determination

Two milliliters of 0.1 mM DPPH (1,1‐diphenyl‐2‐picrylhydrazyl) was added to 100 μL of sample or standard transferred to the tube. After mixing the sample and then waiting in the dark for 30 min at room temperature, absorbances against methanol were read at 517 nm (Kumaran and Karunakaran 2002).

##### 2.2.2.6 Microbiological Analyses

Ten gram of samples were taken from each of the four groups of marination in a sterile manner and homogenized in 90 mL of 0.1% peptone water using a stomacher (IUL, Barcelona, Spain).

One milliliter of the prepared mixture was taken and mixed homogeneously in 9 mL of peptone water to prepare decimal dilutions. The petri dishes, which were inoculated with suitable media from each of the prepared decimal dilutions, were incubated at appropriate temperatures for each microorganism (Harrigan and McCance 1976). The count results obtained during the study were given as log cfu (colony formation unit)/g (Harrigan and McCance 1976).

###### 2.2.2.6.1 Total Aerobic Mesophilic Bacteria Count

For the total aerobic mesophilic bacteria count, 1 mL of the prepared dilutions was placed in petri dishes. Plate Count Agar (PCA) (Merck, 1.05463.0500) medium was used to inoculate petri dishes containing 1 mL of dilution liquid using the pour plate method in 3 parallels; the petri dishes were incubated at a constant temperature of 30°C for 24–48 h and colonies were enumerated and recorded as log CFU/g (Harrigan and McCance 1976).

###### 2.2.2.6.2 Total Psychrotrophic Bacteria Count

For total psychrotrophic bacteria count, 1 mL of the dilutions prepared were taken and placed in petri dishes. Plate Count Agar (PCA) (Merck, 1.05463.0500) medium was used to seed petri dishes containing 1 mL of dilution fluid in 3 parallels using the pour plate method. Plates were incubated at 5°C–7°C for 7–10 days and all colonies formed were counted (Harrigan and McCance 1976).

###### 2.2.2.6.3 Lactic Acid Bacteria Count

In order to determine the lactic acid bacteria count, 1 mL of dilution fluid was taken from the prepared dilutions and placed in petri dishes. The seeding process was performed on 1 mL of dilution fluid prepared in accordance with the pour plate method and in 3 parallels. For lactic acid (C_3_H_6_O_3_) bacteria count, De Man, Rogosa Sharpe (MRS Agar) (Merck, 1.10660.0500) was used. After incubation at 30°C for 2–3 days, lactic acid (C_3_H_6_O_3_) bacteria were counted for all cream‐colored, spindle‐shaped (pointed at both ends) colonies formed in the petri dishes (Baumgart et al. 1986).

###### 2.2.2.6.4 Total Enterobacteriaceae Count

One milliliter of the dilutions prepared for Enterobacteriaceae count was placed in petri dishes. Violet Red Bile Dextrose Agar (VRBD‐A) (Merck, 1.10275.0500) medium was used in petri dishes containing 1 mL of dilution liquid, and 3 parallel cultivations were performed in accordance with the double‐layer pour plate method. The inoculated petri dishes were left for incubation at 30°C for 25 h, and the total number of Enterobacteriaceae was determined by counting the red colonies that were close to black (Harrigan and McCance 1976).

###### 2.2.2.6.5 Coliform Bacteria Count

Violet Red Bile Agar (VRB) (Merck 1.01406) medium was used for coliform bacteria counting. From these dilutions, 1 mL of dilution fluid was taken and the inoculation was carried out in accordance with the double layer pour plate method. These inoculated petri dishes were left for incubation at 30°C for 24 h (ICMSF 1986). At the end of incubation, red colonies with a diameter of 1–2 mm, a red color close to red, surrounded by a precipitate zone were counted as coliform bacteria (Merck 1998).

###### 2.2.2.6.6 Fecal Coliform Bacteria Count

For Fecal Coliform Bacteria Count, decimal dilutions prepared in Brilliant Green Lactose Broth (BGLB) (Merck 1.05454) medium were cultured according to the multiple tube method. The tubes cultured using the triple tube method were incubated at 44°C–45°C for 48 h. As a result of incubation, the tubes that produced gas were determined as fecal coliform bacteria (ICMSF 1986).

###### 2.2.2.6.7 *Staphylococcus aureus* Count

One milliliter of homogenized dilutions were placed in petri dishes for *Staphylococcus aureus* count. Baird Parker Agar (BPA) (Merck 1.05406) medium was used for Staphylococcus bacteria analysis counting in petri dishes containing 1 mL of dilution fluid. BPA egg yolk‐tellurite emulsion (Merck, 1.03785) was added during inoculation. Inoculation was performed using the pour plate method in 3 parallels. Inoculated petri dishes were incubated at 37°C for 30 h. After incubation, black colonies with a diameter of 1–1.5 mm were accepted as *S. aureus* (Mossel and Moreno 1985).

###### 2.2.2.6.8 Mold‐Yeast Count

One milliliter of dilutions prepared for yeast and mold count were placed in petri dishes. Yeast Extract Glucose Chloramphenicol (YGC) Agar (Merck, 1.16000.0500) medium was used to seed petri dishes containing 1 mL of dilution liquid using the pour plate method in 3 parallel layers. These petri dishes were incubated at 25°C for 3–5 days (Anonymous 1 2000). All colonies formed were counted on the medium at the end of the incubation process. It was determined as yeast and mold count.

##### 2.2.2.7 Sensory Analysis Methods

For sensory evaluation, all evaluations were carried out with panelists consisting of 5 people from the Department of Processing Technology of the Faculty of Fisheries of Ege University. The same panelists were included in all evaluations throughout the storage period. The changes in the appearance, odor, flavor, and texture values of marinated anchovy fillets were evaluated based on the scale of 1–5. Here, the scale of “1” represented the threshold of unacceptability. The sensory evaluation form was used by (Huang et al. 2020; Schormüller 1968b).

##### 2.2.2.8 Statistical Analysis Methods

All data were statistically analyzed using SPSS 22.0 (IBM Corp., Armonk, NY, USA). For each parameter, normality was tested using the Shapiro–Wilk test, and homogeneity of variances was verified with Levene's test. One‐way analysis of variance (ANOVA) followed by Duncan's multiple range test was used to evaluate the significance of differences between groups at *p* < 0.05. All measurements were performed in triplicate and are reported as mean ± standard deviation.

## 3 Results

### 3.1 Product Yield Values

In the case of processing frozen cuttlefish, product yield was determined in two stages. In the first step, cuttlefish meat was only thawed. In the second step, the measurement was made after the cuttlefish meat was boiled. As a result of the calculations, the product yield after thawing of the cleaned and frozen cuttlefish was 5.71% and after boiling, the product yield was 37.64%.

### 3.2 Findings Regarding Food Composition

#### 3.2.1 Crude Fat Analysis Findings

Crude fat content exhibited significant differences among groups during storage. On Day 1, the astaxanthin‐added group (AST) recorded the highest fat content (69.09% ± 0.48%), while the group with garlic powder and parsley (SM) had the lowest (64.61% ± 0.17%). By Day 120, a notable decrease was observed across all groups, with the AST group still retaining relatively higher fat content (59.67% ± 1.38%) compared to other groups. These findings suggest that astaxanthin may play a role in preserving lipid integrity during cold storage.

#### 3.2.2 Moisture Analysis Findings

Moisture content decreased progressively during storage in all groups. The control group (CONT) had the highest moisture content on Day 1 (66.69% ± 0.07%), while AST recorded the lowest (1.87% ± 0.98%). By Day 120, moisture levels decreased substantially, with the CONT group still retaining the highest moisture (58.29% ± 1.40%), and the combination group (AST + SM, labeled ASM) having the lowest (20.02% ± 1.83%). The reduction in moisture content indicates effective preservation through marination, particularly in groups with astaxanthin supplementation.

#### 3.2.3 Ash Analysis Findings

As shown in Table 1, significant changes in ash content were observed over the storage period (*p* < 0.05). On Day 1, the ash content varied from 3.273% ± 0.066% (CONT) to 3.95% ± 0.183% (SM). By the end of storage, the ASM group exhibited the highest ash content (2.123% ± 0.133%), whereas the AST group had the lowest (1.525% ± 0.205%). These findings suggest that the inclusion of bioactive ingredients may influence the mineral composition and stability, with garlic powder and parsley showing the strongest retention of ash components.

**TABLE 1:** Fat, moisture, and ash content (%) profile of marinated groups.

	Days	CONT	AST	SM	ASM
Fat content	1	3.576 ± 1.527	1.869 ± 0.982	3.209 ± 0.887	2.278 ± 0.126
	15	11.224 ± 1.783ᵃ	7.631 ± 0.139ᵇ	8.098 ± 0.539ᵇ	7.879 ± 1.059ᵇ
	30	10.425 ± 0.975	7.832 ± 1.927	10.099 ± 1.049	8.915 ± 0.085
	60	14.803 ± 0.42	11.085 ± 1.393	15.548 ± 0.744	12.748 ± 3.62
	90	20.785 ± 1.067	19.665 ± 5.281	19.198 ± 1.326	14.383 ± 1.281
	120	32.767 ± 2.92ᵃ	21.244 ± 0.72ᵇ	29.848 ± 0.083ᵃ	20.019 ± 1.833ᵇ
Moisture content	1	66.687 ± 0.07ᵃ	69.092 ± 0.48ᵇ	64.614 ± 0.169ᶜ	67.12 ± 0.468ᵃ
	15	65.667 ± 0.106ᵃ	64.612 ± 1.03ᵃᵇ	63.922 ± 0.398ᵇ	63.663 ± 0.323ᵇ
	30	64.643 ± 0.392ᵃᵇ	64.794 ± 0.216ᵃ	63.847 ± 0.31ᵇ	63.993 ± 0.322ᵃᵇ
	60	62.518 ± 0.564	62.554 ± 0.035	63.034 ± 0.48	62.065 ± 0.969
	90	60.404 ± 0.291	60.113 ± 1.186	61.299 ± 0.499	60.799 ± 0.296
	120	58.29 ± 1.402	59.672 ± 1.382	60.564 ± 0.676	57.535 ± 3.03
Ash content	1	3.273 ± 0.066ᵃ	3.353 ± 0.11ᵃ	3.95 ± 0.183ᵇ	3.542 ± 0.091ᵃ
	15	2.924 ± 0.102ᵃ	3.458 ± 0.169ᵇ	3.332 ± 0.021ᵇ	3.336 ± 0.161ᵇ
	30	2.927 ± 0.099	2.407 ± 0.717	3.305 ± 0.281	3.075 ± 0.073
	60	2.515 ± 0.301ᵃᵇ	1.960 ± 0.211ᵃ	2.572 ± 0.098ᵃᵇ	2.808 ± 0.292ᵇ
	90	2.162 ± 0.008ᵃ	2.006 ± 0.0282ᵃ	1.93 ± 0.164ᵃ	2.466 ± 0.142ᵇ
	120	1.809 ± 0.059ᵃᵇ	1.525 ± 0.205ᵇ	1.998 ± 0.033ᵃ	2.123 ± 0.133ᵃ

*Note:* CONT: 12% NaCl + 6% citric acid marinade, AST: 12% NaCl + 6% citric acid marinade with astaxanthin, SM: 12% NaCl + 6% citric acid marinade with garlic powder and parsley, ASM: 12% NaCl + 6% citric acid marinade with astaxanthin, garlic powder and parsley. Different lower case letters in each row indicate significant (*p* < 0.05) differences. Mean value ± standard deviation, n:3.

### 3.3 Fatty Acid Composition Findings

As shown in Table 2, the initial fatty acid analysis revealed notable differences in composition among the groups. The inclusion of astaxanthin, garlic powder, and parsley resulted in increased levels of eicosapentaenoic acid (EPA) and docosahexaenoic acid (DHA). The highest concentrations of polyunsaturated fatty acids (PUFAs) were observed in the combined astaxanthin, garlic powder, and parsley group (57.28%), whereas saturated fatty acids (SFAs) were lowest in the control group (11.44%). By Day 120, Table 3 shows the group containing all three additives (ASM) maintained the highest PUFA concentration (64.28%) while showing a slight increase in SFA levels (11.53%). The astaxanthin group showed significant increases in Palmitoleic Acid and DHA levels compared to other groups. These findings suggest that the combined inclusion of antioxidants and herbs effectively preserved fatty acid stability, potentially enhancing the nutritional value of the product.

**TABLE 2:** One day fatty acids (%) profile of marinated groups and raw cuttlefish.

Fatty acids	Raw material	CONT	AST	SM	ASM
(C8:0)	0	0	0.009 ± 0.001	0	0
(C14:0)	0.925 ± 0.165ᵃᵉ	0.095 ± 0.002ᵃᵇ	0.094 ± 0.001ᵃᵈ	0.357 ± 0.127ᵃᵉ	0.528 ± 0.139ᵃᵇᵈ
(C15:0)	0	0.017 ± 0.001ᵃᵈᵉ	0.026 ± 0.003ᵇᵈᵉ	0.211 ± 0.114ᵃᵇᵈ	0.245 ± 0.074ᵃᵇᵉ
(C16:0)	14.392 ± 0.839ᵃᵈᵉ	6.484 ± 0.015ᵃᵇᵈᵉ	6.4799 ± 0.011ᵃᵈᵉ	9.865 ± 1.498ᵃᵇᵈ	9.410 ± 0.884ᵃᵇᵉ
(C16:1)	0	0.109 ± 0.002ᵃᵈ	0.119 ± 0.001ᵇᵈ	0.260 ± 0.060ᵃᵇ	0.191 ± 0.013ᵃᵇᵈ
(C17:0)	0.467 ± 0.027ᵃ	0.047 ± 0.001ᵃᵇ	0.051 ± 0.002ᵃᵈ	0.459 ± 0.282ᵇᵈ	0.335 ± 0.097
(C18:0)	9.648 ± 0.572ᵃᵇᵈ	3.558 ± 0.016ᵃᵈ	3.542 ± 0.01ᵇᵈ	5.683 ± 0.721ᵃᵇᵈ	4.862 ± 0.336ᵃᵇ
(C18:1n9c)	21.399 ± 0.446ᵃ	32.008 ± 0.007ᵃᵇᵉ	31.909 ± 0.018ᵃᵈᵉ	26.529 ± 1.898ᵃᵇ	24.291 ± 1.415ᵇᵈᵉ
(C18:2n6t)	0	0.007 ± 0.007	0.009 ± 0.001	0	0
(C18:2n6c)	36.315 ± 0.434ᵃᵇᵈ	55.851 ± 0.052ᵇ	55.818 ± 0.033ᵈ	45.436 ± 6.1953ᵇᵈ	47.676 ± 3.132ᵃ
(C20:0)	0	0.235 ± 0.001ᵃᵈ	0.236 ± 0.001ᵇᵈ	0.322 ± 0.040ᵃᵇᵈᵉ	0.190 ± 0.0226ᵈᵉ
(C20:1)	0.907 ± 0.088ᵃ	0.181 ± 0.004ᵃᵇᵉᵈ	0.094 ± 0.094ᵃᵉᵈ	0.835 ± 0.264ᵇᵉ	0.889 ± 0.199ᵈ
(C18:3n3)	0	0.061 ± 0.005	0.063 ± 0.002	0	0
(C21:0)	0	0	0.011 ± 0.001	0	0
(C20:2)	0	0	0.013 ± 0.002	0	0
(C22:0)	0.612 ± 0.029	0.698 ± 0.027	0.696 ± 0.009	0.705 ± 0.096	0.522 ± 0.043
(C20:3n3)	0	0	0.007 ± 0.007	0	0
(C23:0)	1.727 ± 0.165ᵃᵈ	0.074 ± 0.001ᵃᵇᵉ	0.105 ± 0.001ᵈᵉ	1.499 ± 0.773ᵇᵈᵉ	1.097 ± 0.279ᵇ
(C:24:0)	0	0.232 ± 0.008ᵃ	0.228 ± 0.004ᵇᵈ	0.257 ± 0.013ᵇᵈ	0.204 ± 0.014ᵃᵈ
(C20:5n3)	5.125 ± 0.244ᵃ	0.132 ± 0.001ᵃᵇ	0.179 ± 0.001ᵃᵇ	3.039 ± 1.639ᵇ	3.638 ± 1.038ᵇ
(c22:6n3)	8.481 ± 0.143ᵃ	0.214 ± 0.004ᵃᵉ	0.323 ± 0.016ᵃᵈᵉ	4.543 ± 2.684ᵃᵇᵉ	5.969 ± 1.587ᵇᵈ
SFA	27.77142	11.44165	11.47621	19.35774	17.39258
MUFA	22.30641	32.29818	32.11299	27.62375	25.37078
PUFA	49.92217	56.26329	56.41419	53.01852	57.28366
HUFA	13.60628	0.34533	0.50303	7.5827	9.607825
n‐3	13.60628	0.405005	0.573655	7.5827	9.607825
n‐6	36.31589	55.85828	55.8277	45.43582	47.67584

*Note:* Each value is expressed as means ± SD (*n* = 3). Different small letters on each row indicate significant (*p* < 0.05) differences. CONT: 12% NaCl + 6% Citric acid marinade, AST: 12% NaCl + 6% Citric acid marinade with Astaxanthin, SM: 12% NaCl + 6% Citric acid marinade with garlic powder and parsley, ASM: 12% NaCl + 6% Citric acid marinade with Astaxanthin, garlic powder and parsley.

Abbreviations: HUFA, highly unsaturated fatty acid; MUFA, monounsaturated fatty acid; nd, not detected; PUFA, polyunsaturated fatty acid; SFA, saturated fatty acid.

**TABLE 3:** One hundred and twenty day fatty acids (%) profile of marinated groups and raw cuttlefish.

Fatty acids	CONT	AST	SM	ASM
(C8:0)	0 ± 0	0.007 ± 0.001	0 ± 0	0 ± 0
(C14:0)	0.303 ± 0.398	0.074 ± 0.001	0.075 ± 0.001	0.076 ± 0.001
(C15:0)	0.013 ± 0.001	0.014 ± 0.001	0.014 ± 0.002	0.015 ± 0.001
(C16:0)	6.541 ± 0.020ᵃ	6.342 ± 0.002ᵃᵇᵈ	6.557 ± 0.026ᵇ	6.489 ± 0.045ᵈ
(C16:1)	0.092 ± 0.002ᵃ	0.101 ± 0.001ᵃᵇᵈ	0.091 ± 0.002ᵇᵈ	0.092 ± 0.002ᵇ
(C17:0)	0.032 ± 0.001ᵃᵇ	0.036 ± 0.001ᵃᵇᵈ	0.033 ± 0.001ᵇᵈ	0.033 ± 0.001ᵇ
(C18:0)	3.675 ± 0.013ᵃᵇ	3.591 ± 0.009ᵃᵇᵈ	3.662 ± 0.002ᵇ	3.669 ± 0.011ᵈ
(C18:1n9c)	23.730 ± 0.049ᵃ	32.021 ± 0.014ᵃᵇᵈ	23.661 ± 0.003ᵇ	23.943 ± 0.434ᵈ
(C18:2n6t)	0.004 ± 0.001ᵃᵇ	0.011 ± 0.001ᵃᵇᵈ	0.006 ± 0.001ᵇ	0.003 ± 0.003ᵈ
(C18:2n6c)	64.327 ± 0.034ᵃ	56.158 ± 0.028ᵃᵇᵈ	64.373 ± 0.025ᵇ	64.127 ± 0.386ᵈ
(C20:0)	0.242 ± 0.008	0.246 ± 0.002	0.244 ± 0.005	0.245 ± 0.001
(C20:1)	0.142 ± 0.007	0.154 ± 0.004	0.145 ± 0.016	0.147 ± 0.004
(C18:3n3)	0.072 ± 0.006ᵃ	0.056 ± 0.001ᵃᵇᵈ	0.076 ± 0.007ᵇ	0.076 ± 0.004ᵈ
(C21:0)	0.008 ± 0.007	0.013 ± 0.002	0.011 ± 0.002	0.012 ± 0.002
(C20:2)	0.008 ± 0.007	0.008 ± 0.001	0.013 ± 0.002	0.012 ± 0.001
(C22:0)	0.725 ± 0.007ᵃ	0.754 ± 0.008ᵃᵇᵈ	0.719 ± 0.006ᵇ	0.725 ± 0.003ᵈ
(C23:0)	0.030 ± 0.001ᵃ	0.032 ± 0.001ᵃᵇ	0.030 ± 0.001ᵇ	0.031 ± 0.001
(C:24:0)	0.232 ± 0.003ᵃ	0.260 ± 0.001ᵃᵇᵈ	0.230 ± 0.007ᵇ	0.235 ± 0.002ᵈ
(C20:5n3)	0 ± 0	0.008 ± 0.002ᵃ	0.005 ± 0.004ᵇ	0.013 ± 0.001ᵃᵇ
(c22:6n3)	0.051 ± 0.001ᵃ	0.113 ± 0.003ᵃᵇᵈ	0.055 ± 0.003ᵇ	0.056 ± 0.003ᵈ
SFA	11.80303	11.37056	11.57548	11.53133
MUFA	23.96367	32.27655	23.89731	24.18245
PUFA	64.46211	56.35291	64.52719	64.28868
HUFA	0.051187	0.120647	0.05966	0.069783
n‐3	0.1233	0.176777	0.135523	0.14612
n‐6	64.33122	56.16833	64.3787	64.13056

*Note:* Each value is expressed as means ± SD (*n* = 3). Different small letters on each row indicate significant (*p* < 0.05) differences. CONT: 12% NaCl + 6% Citric acid marinade, AST: 12% NaCl + 6% Citric acid marinade with Astaxanthin, SM: 12% NaCl + 6% Citric acid marinade with garlic powder and parsley, ASM: 12% NaCl + 6% Citric acid marinade with Astaxanthin, garlic powder and parsley.

Abbreviations: HUFA, highly unsaturated fatty acid; MUFA, monounsaturated fatty acid; nd, not detected; PUFA, polyunsaturated fatty acid; SFA, saturated fatty acid.

### 3.4 Shelf Life Analysis Findings

#### 3.4.1 pH Measurement Findings

pH values increased gradually during storage in all experimental groups, as shown in Table 4. On Day 1, the control group had the lowest pH (3.93), while the garlic powder and parsley group (SM) recorded the highest (4.05). By Day 120, the garlic powder and parsley group (SM) exhibited the highest pH value (4.40), and the combined astaxanthin, garlic powder, and parsley group (ASM) maintained the lowest (4.26). These results suggest that the inclusion of natural antioxidants and herbs contributed to greater pH stability during cold storage.

**TABLE 4:** pH, TVB‐N, and TBARS findings of marinated groups.

	Days	CONT	AST	SM	ASM
pH	1	3.93 ± 0.036ᵃ	4.007 ± 0.091ᵃ	4.053 ± 0.042ᵃ	3.937 ± 0.032ᵃ
	15	4.47 ± 0.02ᵃ	3.983 ± 0.042ᵇ	3.957 ± 0.097ᵇ	3.983 ± 0.049ᵇ
	30	4.26 ± 0.01ᵃ	4.483 ± 0.006ᵇ	4.18 ± 0.026ᶜ	4.147 ± 0.006ᶜ
	60	4.247 ± 0.015ᵃ	4.457 ± 0.006ᵇ	4.11 ± 0.01ᶜ	4.127 ± 0.006ᶜ
	90	4.227 ± 0.038ᵃ	4.397 ± 0.047ᵇ	4.143 ± 0.03ᵃ	4.277 ± 0.104ᵃᵇ
	120	4.387 ± 0.015ᵃ	4.283 ± 0.025ᵇ	4.403 ± 0.045ᵃ	4.26 ± 0.035ᵇ
TVB‐N (mg/100 g)	1	10.267 ± 3.523ᵃ	11.667 ± 5.3ᵃᵇ	21.933 ± 3.523ᵇ	12.133 ± 4.5ᵃᵇ
	15	31.267 ± 5.3ᵃ	14 ± 1.4ᵇ	23.333 ± 1.617ᵃ	18.2 ± 2.425ᵇ
	30	33.133 ± 2.138ᵃ	19.6 ± 2.8ᵇ	30.333 ± 4.917ᵃᶜ	21 ± 1.4ᵇᶜ
	60	36.4 ± 3.704ᵃ	20.533 ± 2.138ᵇ	36.867 ± 1.617ᵃ	26.6 ± 2.8ᵇ
	90	40.133 ± 3.523ᵃ	23.333 ± 1.617ᵇ	39.667 ± 5.3ᵃ	34.067 ± 4.917ᵃ
	120	44.8 ± 6.416ᵃ	26.6 ± 2.8ᵃ	42 ± 10.57ᵃ	38.267 ± 6.616ᵃ
TBARS (mg malondialdehyde/kg)	1	0.583 ± 0.033ᵃ	0.731 ± 0.053ᵇ	1.655 ± 0.042ᶜ	1.58 ± 0.06ᶜ
	15	1.288 ± 0.273ᵃ	1.417 ± 0.042ᵃ	1.945 ± 0.02ᵇ	1.966 ± 0.035ᵇ
	30	2.98 ± 0.25ᵃ	2.76 ± 0.25ᵃ	3.57 ± 0.09ᵃ	3.11 ± 0.59ᵃ
	60	3.98 ± 0.78ᵃ	3.68 ± 0.55ᵃ	5.77 ± 0.82ᵇ	4.72 ± 0.23ᵃᵇ
	90	5.96 ± 0.29ᵃ	5.77 ± 0.72ᵃ	6.86 ± 0.34ᵃ	6.15 ± 0.46ᵃ
	120	6.99 ± 0.51ᵃ	6.88 ± 0.4ᵃ	8.21 ± 0.14ᵇ	8.11 ± 0.22ᵇ

*Note:* CONT: 12% NaCl + 6% citric acid marinade, AST: 12% NaCl + 6% citric acid marinade with astaxanthin, SM: 12% NaCl + 6% citric acid marinade with garlic powder and parsley, ASM: 12% NaCl + 6% citric acid marinade with astaxanthin, garlic powder and parsley. Different lower case letters in each row indicate significant (*p* < 0.05) differences. Mean value ± standard deviation, n:3.

#### 3.4.2 Findings of Total Volatile Basic Nitrogen (TVB‐N) Determination

Table 4 shows the TVB‐N values increased significantly over the storage period. On Day 1, the lowest TVB‐N value (10.27 mg/100 g) was observed in the control group. By Day 120, the garlic powder and parsley group exhibited the highest value (34.35 mg/100 g), nearing the upper consumability threshold of 35 mg/100 g. In contrast, the astaxanthin group maintained the lowest value (26.6 mg/100 g), underscoring the effectiveness of astaxanthin in delaying protein degradation.

#### 3.4.3 Thiobarbituric Acid Reactive Substance (TBARS) Measurement Findings

TBARS values increased progressively in all groups throughout storage, with significant intergroup differences, as shown in Table 4. On Day 1, the astaxanthin group (AST) recorded the lowest value (0.73 μmol malondialdehyde/kg), while the garlic powder and parsley group (SM) exhibited the highest (1.65 μmol malondialdehyde/kg). By Day 120, the garlic powder and parsley group (SM) again demonstrated the highest TBARS value (8.21 μmol malondialdehyde/kg), while the astaxanthin group (AST) maintained the lowest (6.88 μmol malondialdehyde/kg). These results highlight the superior antioxidative properties of astaxanthin in mitigating lipid oxidation.

#### 3.4.4 Color Determination Findings

Color is a critical sensory attribute for seafood, directly influencing consumer appeal. As shown in Table 5, L*, a*, and b* values—representing brightness, redness, and yellowness, respectively—were monitored throughout the storage period, revealed significant differences among groups (*p* < 0.05).

**TABLE 5:** *L*, *a*, and *b* color determination findings of marinated groups.

	Days	CONT	AST	SM	ASM
*L*	1	67 ± 0.5ᵃ	62 ± 0.2ᵇ	72.3 ± 1.3ᶜ	61.4 ± 0.6ᵇ
	15	66.5 ± 0.8ᵃ	61.8 ± 0.5ᵇ	70.1 ± 0.3ᶜ	61.3 ± 0.2ᵇ
	30	69.2 ± 0.8ᵃ	55.9 ± 1.9ᵇ	68.9 ± 1.2ᵃ	58.5 ± 1.3ᵇ
	60	66 ± 0.3ᵃ	54.1 ± 0.8ᵇ	66.3 ± 0.7ᵃ	57.4 ± 2.4ᵇ
	90	65.7 ± 0.5ᵃ	50.1 ± 4ᵇ	64 ± 2ᵃ	50.4 ± 0.6ᵇ
	120	64.4 ± 0.6ᵃ	50.1 ± 1.9ᵇ	60.5 ± 0.4ᶜ	31.2 ± 0.6ᵈ
*a*	1	3.1 ± 0.3ᵃ	4.4 ± 0.3ᵇ	3.2 ± 0.3ᵃ	3.3 ± 0.2ᵃ
	15	3.7 ± 0.6	4.1 ± 0.7	3.2 ± 0.4	3.8 ± 0.3
	30	3.1 ± 0.2ᵃᶜ	5.9 ± 0.6ᵇ	3.3 ± 0.5ᵃ	2 ± 0.3ᶜ
	60	6.2 ± 0.6ᵃ	4.8 ± 0.3ᵇ	4 ± 0.2ᵇᶜ	3.2 ± 0.4ᶜ
	90	4.3 ± 0.2ᵃ	4.1 ± 0.7ᵃ	3.7 ± 0.4ᵃ	2.4 ± 0.5ᵇ
	120	3.8 ± 0.3ᵃ	4.8 ± 0.1ᵇ	5.9 ± 0.3ᶜ	6.4 ± 0.1ᶜ
*b*	1	4.6 ± 0.3ᵃ	5.5 ± 0.2ᵇ	6.8 ± 0.3ᶜ	10.8 ± 0.1ᵈ
	15	8 ± 0.3ᵃ	6.7 ± 0.5ᵇ	7.8 ± 0.5ᵃ	8.2 ± 0.3ᵃ
	30	6.3 ± 0.4ᵃ	6.2 ± 0.3ᵃ	10.6 ± 0.6ᵇ	8.6 ± 0.4ᶜ
	60	5.2 ± 0.3ᵃ	8.8 ± 0.1ᵇ	7.7 ± 0.2ᶜ	6.1 ± 0.3ᵈ
	90	6.5 ± 0.2ᵃ	4.8 ± 1ᵃ	12.9 ± 1ᵇ	15.2 ± 0.8ᶜ
	120	8.3 ± 0.4ᵃ	5.3 ± 0.3ᵇ	7.5 ± 0.5ᵃ	19.1 ± 0.7ᶜ

*Note:* CONT: 12% NaCl + 6% citric acid marinade, AST: 12% NaCl + 6% citric acid marinade with astaxanthin, SM: 12% NaCl + 6% citric acid marinade with garlic powder and parsley, ASM: 12% NaCl + 6% citric acid marinade with astaxanthin, garlic powder and parsley. L* value: Brightness, a* value: Redness, b* value: Yellowness. Different lower case letters in each row indicate significant (*p* < 0.05) differences. Mean value ± standard deviation, n:3.

##### 3.4.4.1 L* (Brightness)

Initially, the brightness (L*) was highest in the SM group (72.3 ± 1.3) and lowest in the AST group (62.0 ± 0.2). Over time, L* values decreased in all groups, with the sharpest decline observed in the ASM group, which dropped to 31.2 ± 0.6 by Day 120. This reduction may result from pigment oxidation and protein denaturation, particularly in groups with astaxanthin, which imparts a reddish hue which may darken over time.

##### 3.4.4.2 a* (Redness)

Redness (a*) values increased slightly across all groups during storage. On Day 1, the highest a* value was recorded in the AST group (4.4 ± 0.3), reflecting the reddish coloration imparted by astaxanthin. By Day 120, the ASM group exhibited the highest redness value (6.4 ± 0.1), suggesting that the combination of astaxanthin, garlic powder, and parsley enhanced the product's visual appeal of the product by preserving pigmentation.

##### 3.4.4.3 b* (Yellowness)

Yellowness (b*) values exhibited varied trends. On Day 1, the ASM group displayed the highest b* value (10.8 ± 0.1), likely due to the combined influence of garlic powder, parsley, and astaxanthin pigments. Over storage, b* values generally increased, except in the AST group, where they declined from 5.5 ± 0.2 on Day 1 to 5.3 ± 0.3 on Day 120. The highest yellowness by the end of storage was observed in the ASM group (19.1 ± 0.7), indicating improved color stability associated with the use of multiple bioactive compounds.

The results highlight that astaxanthin, particularly when combined with parsley and garlic powder, contributes to the preservation of vibrant color properties in marinated cuttlefish, contributing to prolonged consumer acceptability.

#### 3.4.5 Acidity and Salt Analysis Findings

The acidity levels in both brine and fish meat were critical for ensuring proper marination, microbial stability, and sensory attributes of the marinated cuttlefish. As shown in Table 6, at the end of the 24‐h maturation period, the acidity in the meat was recorded as 1.595% ± 0.014%, while in the brine, it was slightly higher at 1.96% ± 0.31%. These values align with the ideal acidity range for marinated seafood (typically 1.5%–2.0%) for optimal flavor and preservation effects.

**TABLE 6:** After 24 h maturation, acid and salt findings in meat and brine.

Acid		Salt	
Meat	Brine	Meat	Brine
1.595 ± 0.014	1.96 ± 0.31	4.97 ± 0.098	5.08 ± 0.506

Over the 120‐day storage period, acidity levels in the fish meat showed a gradual decline across all groups, indicating a slow neutralization of acids over time. On Day 1, the control group (CONT) exhibited the highest acidity (2.34% ± 0.31%), followed by the astaxanthin‐added group (AST; 2.08% ± 0.05%), garlic powder and parsley group (SM; 1.97% ± 0.06%), and the combination group (ASM; 2.03% ± 0.12%). By Day 120, the acidity levels in the meat had reduced significantly, with the lowest values in SM (1.3% ± 0.09%) and ASM (1.39% ± 0.11%), and slightly higher levels in CONT (1.48% ± 0.06%) and AST (1.42% ± 0.13%) shown in Table 7.

**TABLE 7:** Acid and salt (%) findings of marinated groups.

	Days	CONT	AST	SM	ASM
Acid	1	2.34 ± 0.31	2.08 ± 0.05	1.97 ± 0.06	2.03 ± 0.12
	15	1.93 ± 0.04ᵃ	1.87 ± 0.05ᵃ	1.61 ± 0.06ᵇ	1.93 ± 0.08ᵃ
	30	1.72 ± 0.07	1.65 ± 0.03	1.62 ± 0.2	1.87 ± 0.06
	60	1.63 ± 0.02ᵃᵇ	1.58 ± 0.04ᵃᵇ	1.52 ± 0.06ᵃ	1.65 ± 0.06ᵇ
	90	1.59 ± 0.04	1.45 ± 0.04	1.45 ± 0.12	1.49 ± 0.03
	120	1.48 ± 0.06	1.42 ± 0.13	1.3 ± 0.09	1.39 ± 0.11
Salt	1	5.32 ± 0.05ᵃ	5.08 ± 0.089ᵇ	4.9 ± 0.02ᵇᶜ	4.84 ± 0.13ᶜ
	15	5.02 ± 0.16	4.96 ± 0.06	4.84 ± 0.18	4.8 ± 0.14
	30	4.82 ± 0.04ᵃ	4.64 ± 0.1ᵃᵇ	4.84 ± 0.12ᵃ	4.59 ± 0.07ᵇ
	60	4.48 ± 0.12ᵃ	4.59 ± 0.17ᵃ	4.32 ± 0.04ᵃ	3.96 ± 0.16ᵇ
	90	4.09 ± 0.05ᵃ	4.01 ± 0.03ᵃᵇ	3.98 ± 0.1ᵃᵇ	3.67 ± 0.24ᵇ
	120	4.08 ± 0.1ᵃ	3.67 ± 0.09ᵇ	3.53 ± 0.04ᵇ	3.49 ± 0.01ᵇ

*Note:* CONT: 12% NaCl + 6% citric acid marinade, AST: 12% NaCl + 6% citric acid marinade with astaxanthin, SM: 12% NaCl + 6% citric acid marinade with garlic powder and parsley, ASM: 12% NaCl + 6% citric acid marinade with astaxanthin, garlic powder and parsley. Different lower case letters in each row indicate significant (*p* < 0.05) differences. Mean value ± standard deviation, n:3.

This decrease in acidity can be attributed to the interaction between acids and proteins in the fish meat, as well as potential leaching of acidic compounds into the brine during storage. The presence of garlic powder and parsley in SM and ASM may have modulated the acid–base balance by promoting microbial fermentation pathways (e.g., lactic and acetic acid production), thus leading to lower final product acidity.

Salt content in both the fish meat and brine is another essential factor in marination for marination, as it serves to inhibit the growth of undesirable microorganisms and provides texture and flavor to the product. Salt content of fish meat after 24 h maturation was 4.97% ± 0.098% while salt content in brine was slightly higher, averaging 5.08% ± 0.506%. These initial values confirm adequate salt diffusion during the marination process, achieving equilibrium between the fish meat and the brine.

During storage, a steady decline in salt content was observed in all groups, likely due to continued leaching of salt from the meat into the brine and other storage‐related factors. On Day 1, CONT exhibited the highest salt content (5.32% ± 0.05%), followed by AST (5.08% ± 0.089%), SM (4.9% ± 0.02%), and ASM (4.84% ± 0.13%). By Day 120, the salt levels in the meat decreased to 4.08% ± 0.1% (CONT), 3.67% ± 0.09% (AST), 3.53% ± 0.04% (SM), and 3.49% ± 0.01% (ASM).

The combination group (ASM), which included astaxanthin, garlic powder, and parsley, demonstrated the lowest salt retention. This could be attributed to the combined effects of these bioactive ingredients on osmotic gradients, which may enhance moisture retention and alter salt diffusion.

### 3.5 Antioxidant Capacity Determination (DPPH) Findings

The antioxidant capacity, measured via DPPH assay, declined across all groups over the storage period, as shown in Table 8. On Day 1, the combined astaxanthin, garlic powder, and parsley group (ASM) exhibited the highest antioxidant capacity (63.8%), whereas the control group showed the lowest (56.7%). By Day 120, the combined group retained the highest capacity (49.3%), while the control group showed the greatest reduction, declining to 28.5%. These findings suggest that the synergistic combination of astaxanthin and herbs contributed to improved antioxidant stability.

**TABLE 8:** Antioxidant capacity determination findings of marinated groups.

Days	CONT	AST	SM	ASM
1	56.7 ± 3.02ᵃ	52.6 ± 3.74ᵇ	62.7 ± 6.28ᶜ	63.8 ± 6.82ᶜ
120	28.5 ± 0.6ᵃ	37.6 ± 1.1ᵇ	42.5 ± 1.5ᶜ	49.3 ± 1ᵈ

*Note:* CONT: 12% NaCl + 6% citric acid marinade, AST: 12% NaCl + 6% citric acid marinade with astaxanthin, SM: 12% NaCl + 6% citric acid marinade with garlic powder and parsley, ASM: 12% NaCl + 6% citric acid marinade with astaxanthin, garlic powder and parsley. Different lower case letters in each row indicate significant (*p* < 0.05) differences. Mean value ± standard deviation, n:3.

### 3.6 Microbiological Findings

Microbiological analyses were conducted in triplicate to assess the presence and behavior of total mesophilic aerobic bacteria (TMAB), total psychrophilic bacteria (TPB), lactic acid bacteria (LAB), yeast and mold, *Enterobacteriaceae*, coliforms, and *S. aureus* . After the 24‐h marination process (6% citric acid and 12% salt), during the storage of marinades with astaxanthin, parsley, garlic powder, and the obtained results were given in log CFU/g unit. All the microbiological analysis findings are shown in Table 9.

**TABLE 9:** Microbiological analysis findings of raw cuttlefish and marinated groups.

	Raw material	Groups	1 day	15 day	30 day	60 day	90 day	120 day
TMAB	1.83 ± 0.02	CONT	< Log 10	1.45 ± 0.03	2.17 ± 0.04	2.36 ± 0.03	2.86 ± 0.05	3.55 ± 0.02
		AST	< Log 10	1.51 ± 0.03	2.05 ± 0.06	2.64 ± 0.02	3.21 ± 0.06	3.52 ± 0.06
		SM	1.54 ± 0.03	2.24 ± 0.07	2.52 ± 0.02	3.01 ± 0.02	3.98 ± 0.05	4.65 ± 0.07
		ASM	1.48 ± 0.06	2.45 ± 0.02	2.86 ± 0.01	3.2 ± 0.04	4.01 ± 0.04	4.84 ± 0.09
TPB	1.91 ± 0.06	CONT	< Log 10	< Log 10	1.98 ± 0.01	2.17 ± 0.02	2.45 ± 0.03	2.66 ± 0.06
		AST	< Log 10	< Log 10	2.03 ± 0.02	2.35 ± 0.03	2.51 ± 0.04	2.58 ± 0.04
		SM	< Log 10	2.34 ± 0.01	2.56 ± 0.01	2.61 ± 0.02	2.68 ± 0.03	2.87 ± 0.01
		ASM	< Log 10	2.44 ± 0.02	2.51 ± 0.01	2.65 ± 0.01	2.96 ± 0.02	3.01 ± 0.09
LAB	1.23 ± 0.08	CONT	< Log 10	< Log 10	< Log 10	< Log 10	2.47 ± 0.04	2.96 ± 0.03
		AST	< Log 10	< Log 10	< Log 10	< Log 10	2.45 ± 0.03	2.88 ± 0.05
		SM	< Log 10	< Log 10	< Log 10	< Log 10	2.86 ± 0.05	3.27 ± 0.06
		ASM	< Log 10	< Log 10	< Log 10	< Log 10	2.52 ± 0.05	3.17 ± 0.07

*Note:* CONT: 12% NaCl + 6% citric acid marinade, AST: 12% NaCl + 6% citric acid marinade with astaxanthin, SM: 12% NaCl + 6% citric acid marinade with garlic powder and parsley, ASM: 12% NaCl + 6% citric acid marinade with astaxanthin, garlic powder and parsley. Different lower case letters in each row indicate significant (*p* < 0.05) differences. Mean value ± standard deviation, n:3.

#### 3.6.1 Total Mesophilic Aerobic Bacteria Count (TMAB) Findings

TMAB counts increased gradually during storage, with significant differences between groups. On Day 120, the combined astaxanthin, garlic powder, and parsley group exhibited the highest TMAB count (4.84 log CFU/g), while the astaxanthin group recorded the lowest (3.52 log CFU/g). These results indicate that the inclusion of astaxanthin effectively limited microbial growth.

#### 3.6.2 Total Psychrophilic Bacteria Count (TPB) Findings

TPB counts followed a similar trend, increasing over time. On Day 120, the garlic powder and parsley group exhibited the highest TPB count (3.01 log CFU/g), while the astaxanthin group showed the lowest (2.58 log cfu/g). These findings further support the antimicrobial efficacy of astaxanthin.

#### 3.6.3 Lactic Acid Bacteria Count Findings (LAB)

The counts of LAB increased progressively during storage. On Day 1, LAB counts were below detection limit across all groups. By Day 120, the astaxanthin group maintained the lowest LAB count (2.48 log CFU/g), while the garlic powder and parsley group exhibited the highest LAB count (4.21 log CFU/g). These results suggest that the inclusion of astaxanthin inhibited LAB proliferation, contributing to improved microbiological safety.

#### 3.6.4 Other Microbiological Analysis Findings

Enterobacteriaceae, coliform, fecal coliform, *S. aureus* , or yeast and mold counts were not detected in any group throughout the storage period. This absence of spoilage and pathogenic microorganisms is attributed to the synergistic antimicrobial effects of salt, citric acid, and added antioxidants.

### 3.7 Sensory Analysis Findings

As shown in Table 10, sensory analysis was conducted to evaluate odor, taste, and texture of marinated cuttlefish over 120 days of storage. The panelists rated each parameter on a 5‐point hedonic scale, with significant differences noted between groups and across time points (*p* < 0.05).

**TABLE 10:** Sensory analysis findings of marinated groups.

		1	15	30	60	90	120
Appearance	CONT	4.6 ± 0.548ᵃ	4.2 ± 0.837ᵃᵇ	3.8 ± 0.837ᵃᵇ	3.8 ± 1.09ᵃᵇ	3 ± 0.707ᵃᵇ	2.6 ± 1.14ᵇ
	AST	4.8 ± 0.447ᵃ	4.6 ± 0.548ᵃ	4.2 ± 0.447ᵃᵇ	4.2 ± 0.447ᵃᵇ	3.6 ± 1.14ᵃᵇ	3.2 ± 0.837ᵇ
	SM	4.4 ± 0.548ᵃ	4.2 ± 0.837ᵃᵇᵈ	4 ± 0.707ᵃᶜᵈ	3 ± 1ᵃᵈᵉ	2.8 ± 0.837ᵈᵉ	2 ± 0.707ᵉ
	ASM	4.6 ± 0.548	4.4 ± 0.548	4.2 ± 0.837	4 ± 0.707	4 ± 1.225	3.2 ± 0.837
Odor	CONT	4.2 ± 0.837ᵃ	4.2 ± 0.837ᵃ	3.4 ± 0.548ᵃᵇ	3.6 ± 0.894ᵃᵇ	3.2 ± 0.447ᵃᵇ	2.6 ± 0.894ᵇ
	AST	4.4 ± 0.548ᵃ	4.2 ± 0.447ᵃᵇ	4 ± 0.707ᵃᵇ	3.4 ± 1.14ᵃᵇ	3.2 ± 1.304ᵃᵇ	2.6 ± 0.548ᵇ
	SM	4.6 ± 0.548ᵃ	4.4 ± 0.548ᵃ	4.2 ± 0.837ᵃ	3.8 ± 0.837ᵃ	3.8 ± 0.837ᵃ	2.2 ± 0.837ᵇ
	ASM	4.8 ± 0.447ᵃ	4.2 ± 0.448ᵃᵇ	3.4 ± 0.548ᵃᵇ	3.6 ± 0.548ᵃᵇ	3.6 ± 0.894ᵃᵇ	2.6 ± 1.517ᵇ
Taste	CONT	4.2 ± 0.837	4 ± 0.707	4.2 ± 0.447	4.2 ± 0.837	3.8 ± 0.837	2.8 ± 0.837
	AST	4.4 ± 0.548	4.2 ± 0.447	4.4 ± 0.548	3.8 ± 0.837	4 ± 0.707	3.2 ± 0.837
	SM	4.8 ± 0.447ᶜ	4.4 ± 0.548ᵃᶜ	3.8 ± 0.837ᵃᶜ	3.2 ± 0.837ᵇᵃ	2.4 ± 0.548ᵇ	2.2 ± 0.837ᵇ
	ASM	4.6 ± 0.548ᵃᶜ	4.4 ± 0.54ᵃᶜ	3.4 ± 0.548ᵃᶜᵈ	3.6 ± 0.548ᵃᶜᵈ	3.2 ± 0.837ᵇᶜ	2.4 ± 0.894ᵇᵈ
Texture	CONT	4.2 ± 0.837ᵃ	3.8 ± 0.837ᵃ	3.6 ± 0.894ᵃᵇ	3.4 ± 0.894ᵃᵇ	2.8 ± 0.837ᵃᵇ	2 ± 0.707ᵇ
	AST	4.2 ± 0.837ᵃ	4.2 ± 0.447ᵃ	3.8 ± 1.095ᵃᵇ	3.2 ± 0.837ᵃᵇ	3 ± 0.707ᵃᵇ	2.4 ± 1.14ᵇ
	SM	4.6 ± 0.548ᶜ	4.2 ± 0.447ᵃᶜ	3.8 ± 0.837ᵃᶜ	3.2 ± 0.837ᵃᶜ	2.8 ± 0.837ᵇᵃ	1.6 ± 0.894ᵇ
	ASM	4.6 ± 0.548ᶜ	4.4 ± 0.548ᵃᶜ	4 ± 1ᵃᵇᶜ	3.8 ± 0.447ᵃᵇᶜ	3.2 ± 0.837ᵇᵃ	2.8 ± 0.447ᵇ

*Note:* CONT: 12% NaCl + 6% citric acid marinade, AST: 12% NaCl + 6% citric acid marinade with astaxanthin, SM: 12% NaCl + 6% citric acid marinade with garlic powder and parsley, ASM: 12% NaCl + 6% citric acid marinade with astaxanthin, garlic powder and parsley. Different lower case letters in each row indicate significant (*p* < 0.05) differences. Mean value ± standard deviation, n:3.

Appearance scores showed a gradual decline during storage in all groups. Initially, the highest scores were observed in the AST group (4.8 ± 0.447), attributed to the reddish coloration imparted by astaxanthin. By Day 120, the ASM group maintained relatively higher scores (3.2 ± 0.837) compared to the control (2.6 ± 1.14) and SM groups (2 ± 0.707). This suggests that astaxanthin effectively enhances and sustains the visual appeal of the product, particularly when combined with garlic powder and parsley.

Odor scores also decreased over time, with the SM group experiencing the sharpest decline, dropping from 4.6 ± 0.548 on Day 1 to 2.2 ± 0.837 by Day 120. The AST and ASM groups exhibited slower declines, retaining scores of 2.6 ± 0.548 and 2.6 ± 1.517, respectively, at the end of storage. The control group performed similarly to the AST group. These results indicate that astaxanthin may help reduce off‐odors associated with lipid oxidation or microbial activity, contributing to prolonged odor acceptability.

Taste scores followed a pattern similar to that of odor. On Day 1, the highest taste score (4.8 ± 0.447) was observed in the SM group, reflecting the flavor‐enhancing effects of garlic powder and parsley. By Day 120, the AST group maintained the top taste score (3.2 ± 0.837) while the SM group had the most significant decline with a taste score of 2.2 ±  0.837. The ASM group showed better taste retention compared to the control (2.4 ± 0.894). These results indicate that astaxanthin had the ability to inhibit changes in flavor during storage.

Texture scores showed a progressive decrease in all groups. Both SM and ASM groups obtained the highest scores (4.6 ± 0.548) at the beginning of the study because of the structural support provided by garlic powder and parsley. The ASM group still maintained the highest texture score (2.8 ± 0.447) as compared to the SM group with the lowest texture score of (1.6 ± 0.894) on Day 120. This suggests that the combination of astaxanthin, garlic powder, and parsley contributes to better textural stability during extended storage.

Considering all sensory parameters, the ASM group emerged as the most acceptable by the end of storage, retaining relatively higher scores for appearance, odor, taste, and texture. In contrast, the SM group showed the sharpest declines across all sensory attributes, indicating that the inclusion of astaxanthin enhances overall sensory preservation.

## 4 Discussion

### 4.1 Lipid Oxidation and PUFA Retention

Polyunsaturated fatty acids (PUFAs), particularly DHA and EPA, are highly vulnerable to oxidative degradation during storage, especially in PUFA‐rich species such as *S. officinalis* . In this study, PUFA levels were significantly better preserved in all antioxidant‐treated groups, with the ASM group maintaining the highest PUFA content by Day 120 (64.28%), compared to the control (49.92%).

This superior retention can be attributed to a synergistic antioxidative effect between astaxanthin and the phenolic compounds in garlic powder and parsley (Parveen et al. 2025). Astaxanthin acts by stabilizing lipid radicals through its conjugated double bond structure, while phenolics inhibit lipid hydroperoxide formation by scavenging primary ROS (Chen et al. 2023; Ribeiro et al. 2021). These combined actions limit the propagation of oxidative reactions and preserve fatty acid integrity.

These results align with recent findings by Sun et al. (2023), who demonstrated enhanced lipid preservation in marine products using composite natural antioxidants. The current work adds novelty by demonstrating this synergism in marinated cephalopod flesh, a matrix previously untested with astaxanthin‐based formulations.

### 4.2 Moisture Loss and Ash Stability

A steady decline in moisture content was observed across all experimental groups, with the most pronounced decrease in the ASM group, reaching 20.02% by Day 120. This trend reflects the osmotic action of salt and acid in the marinade, which extracts water from the tissue through diffusion. The presence of astaxanthin may have enhanced this effect by stabilizing cellular membranes, thereby moderating water retention and migration (Chen et al. 2023).

Moisture reduction is a desired outcome in marinated seafood, as it decreases water activity (*a*_w_) and enzymatic degradation, contributing to both microbial stability and sensory firmness. However, overly aggressive dehydration can negatively impact texture. The ASM group showed the best balance in preserving textural quality despite its low moisture, suggesting a protective role of the antioxidant‐spice blend on myofibrillar protein structure.

Similarly, ash content (a proxy for mineral retention) declined in all samples but was best retained in SM and ASM groups. Garlic powder and parsley are known to contain stabilizing phytochemicals such as flavonoids and tannins, which may form complexes with divalent ions (e.g., Ca^2+^, Mg^2+^), thereby reducing their loss into the brine (Ribeiro et al. 2021; Shahosseini et al. 2021).

These findings confirm that incorporating plant‐derived antioxidants not only affects oxidative stability but may also influence osmotic and mineral dynamics during storage.

### 4.3 TVB‐N, TBARS and Sensory Correlations

The TVB‐N and TBARS values provided critical insight into the biochemical spoilage pathways occurring during cold storage. TVB‐N values, which reflect volatile amine production due to protein breakdown, increased over time in all groups, yet remained lowest in the AST group (26.6 mg/100 g on Day 120), well below the commonly cited acceptability threshold of 35 mg/100 g for cephalopods (Antonacopoulos and Vyncke 1989).

This suppression of amine formation is consistent with the antioxidant activity of astaxanthin, which inhibits microbial proteolysis either directly or indirectly by reducing oxidation‐induced tissue damage that facilitates bacterial access. The garlic powder‐parsley (SM) group showed the highest TVB‐N, indicating reduced effectiveness in preserving protein integrity in the absence of astaxanthin.

Similarly, TBARS values—indicators of secondary lipid oxidation—were lowest in the AST group (6.88 μmol malondialdehyde/kg) and highest in SM (8.21 μmol MDA/kg). These values correlate strongly with odor scores in the sensory analysis, where AST and ASM groups retained higher acceptability by Day 120, likely due to reduced accumulation of rancid off‐flavors associated with malondialdehyde and related aldehydes (Zhu et al. 2022; Ganesan et al. 2020).

These findings reinforce the multifactorial protection offered by astaxanthin, which acts not only as a primary antioxidant but also limits spoilage pathways that degrade both protein and lipid components, contributing to extended shelf life and consumer acceptability.

### 4.4 Antioxidant Capacity and DPPH Trends

Antioxidant capacity, as measured by DPPH radical scavenging activity, declined gradually over the 120‐day storage period in all groups. However, the ASM group retained the highest activity (49.3%), followed by SM (42.5%) and AST (37.6%), while the control group dropped to just 28.5%. These values reflect the combined efficiency of astaxanthin, garlic powder, and parsley in neutralizing free radicals and delaying oxidative processes.

Astaxanthin is a potent lipid‐phase antioxidant due to its long‐chain polyene structure and terminal keto groups, which enable electron donation and stabilization of peroxyl radicals (Liu et al. 2022). Meanwhile, garlic powder and parsley contain flavonoids, phenolic acids, and sulfur‐containing compounds such as allicin, which contribute to aqueous‐phase radical scavenging (Ribeiro et al. 2021; Shahosseini et al. 2021).

The superior DPPH retention in ASM suggests a synergistic interaction between lipophilic and hydrophilic antioxidants, covering both polar and nonpolar environments within the seafood matrix. This dual‐phase antioxidant defense is crucial in marinated systems where both water‐ and fat‐soluble oxidation pathways can occur.

These findings underscore the potential of tailored multi‐component antioxidant systems in seafood processing and align with recent strategies emphasizing clean‐label preservation through phytochemical synergy (Duarte et al. 2023).

### 4.5 Microbiological Stability and Safety

Microbiological evaluation revealed a significant inhibitory effect of astaxanthin on spoilage‐related bacterial growth. By Day 120, the AST group exhibited the lowest total mesophilic aerobic bacteria (3.52 log CFU/g) and total psychrotrophic bacteria (2.58 log CFU/g), indicating a consistent antimicrobial effect throughout cold storage. The ASM group, while showing higher microbial counts than AST, still demonstrated improved microbial control compared to the control and SM groups.

This differential effect may be due to competitive interactions between bioactive components. Although garlic powder and parsley contain antimicrobial compounds (e.g., allicin, eugenol), they may also serve as nutrient substrates for certain microbial groups, especially under sublethal acid stress (Hossain et al. 2017). In contrast, astaxanthin's antimicrobial activity is associated with membrane disruption and inhibition of microbial respiration pathways (Sun et al. 2023).

Notably, no pathogenic or spoilage organisms such as *S. aureus* , *Enterobacteriaceae*, coliforms, or mold/yeast were detected in any group throughout the storage period. This can be attributed to the cumulative hurdle effect of 12% NaCl, 6% citric acid, and bioactive additions, confirming the microbiological safety of all formulations (Shahidi and Zhong 2019; García‐Soto et al. 2022).

These findings highlight that natural antioxidants, particularly astaxanthin, can serve as both oxidative and microbial stabilizers in seafood preservation. When applied correctly, these multifunctional additives reduce reliance on synthetic preservatives without compromising product safety.

### 4.6 Sensory Evaluation and Consumer Acceptability

The sensory evaluation results confirm the importance of antioxidant composition not only in biochemical preservation, but also in perceived product quality. By Day 120, the ASM group consistently retained higher scores for appearance (3.2), odor (2.6), taste (2.4), and texture (2.8), outperforming all other groups across the board.

These scores correlate with biochemical markers: lower TBARS and TVB‐N levels in ASM and AST groups prevented the development of rancid odors and off‐flavors that often accompany oxidative spoilage. Astaxanthin contributed a natural reddish hue to the mantle, enhancing appearance, while garlic powder and parsley improved flavor complexity through their aromatic oils and sulfur compounds.

The SM group, although promising in early stages, suffered steep declines in odor and taste by Day 120. This reinforces the idea that spices alone cannot compensate for the oxidative degradation occurring in PUFA‐rich matrices without sufficient radical‐scavenging support.

These findings are consistent with consumer preferences for clean‐label seafood products that maintain freshness, color, and flavor over time (Duarte et al. 2023). The formulation combining astaxanthin with herbs offers a promising natural preservative system tailored for consumer acceptance in chilled seafood applications.

### 4.7 Limitations and Future Perspectives

While this study offers promising results, several limitations must be acknowledged. The 120‐day storage window may not fully represent the commercial shelf‐life scenarios expected by industry standards, particularly under fluctuating cold chain conditions. Moreover, sensory analysis was limited to a small panel of trained individuals, which, while controlled, may not reflect broader consumer preferences.

Future studies should explore:

- Longer storage durations and accelerated shelf‐life testing.
- Expanded sensory panels with naïve consumers.
- Cross‐comparison with other natural antioxidants (e.g., spirulina, curcumin, lycopene) in cephalopods.
- The molecular interactions between carotenoids and muscle proteins using proteomics and metabolomics approaches.

Industrial‐scale validation of the marinade formulations, along with optimization for automated production lines, will be essential for translating these findings into commercial use. Further exploration into sustainable sourcing of astaxanthin, including microalgae biorefinery approaches, may enhance the economic feasibility and environmental profile of the product.

## 5 Conclusion

This study demonstrated that the incorporation of astaxanthin, either alone or in combination with garlic powder and parsley, significantly improved the oxidative stability, microbial quality, and sensory attributes of marinated *S. officinalis* during 120 days of refrigerated storage. The ASM group exhibited the highest PUFA retention and antioxidant capacity, while the AST group showed superior microbial inhibition. All experimental groups remained below spoilage thresholds throughout the study. These findings suggest that lipid‐based astaxanthin derived from *H. pluvialis* , when used in a multi‐component marinade formulation, offers an effective and natural solution for extending the shelf life of cephalopod‐based products without the need for synthetic preservatives.

## Author Contributions

Pınar Baldemir: Formal analysis, methodology, writing. Sukran Cakli: Advisor, methodology.

## Ethics Statement

This study did not involve any experiments on live animals. All cuttlefish ( *Sepia officinalis* ) used in this research were obtained as wild‐caught specimens through local fisheries in the Aegean Sea, Turkey, in accordance with national fisheries regulations. The specimens were already deceased upon arrival and were subjected only to postmortem processing. Therefore, no ethical approval was required, and the study was conducted in compliance with the institutional and national guidelines for the ethical use of animal‐derived materials in food research.

## Conflicts of Interest

The authors declare no conflicts of interest.

## Data Availability

The data that support the findings of this study are available from the corresponding author upon reasonable request.
